# Exploring urban male non-marital sexual behaviours in Pakistan

**DOI:** 10.1186/1742-4755-10-22

**Published:** 2013-04-11

**Authors:** Ali M Mir, Abdul Wajid, Stephen Pearson, Mumraiz Khan, Irfan Masood

**Affiliations:** 1 Population Council, No. 7, Street 62, F-6/3, Islamabad, Pakistan; 2 Leeds Institute of Health Sciences, University of Leeds, Room G23, Charles Thackrah Building, 101 Clarendon Road, Leeds, LS2 9LJ, UK

**Keywords:** Behaviours, Men, Urban, Pakistan

## Abstract

**Background:**

In Pakistan, sexual practices outside marriage are proscribed by law. We aimed to assess the range and magnitude of non-marital sexual behaviours of urban men, focusing on men having sex with men.

**Methods:**

In this cross sectional survey undertaken in six cities of Pakistan, we interviewed 2400 men aged 16–45 years selected through a multistage systematic sampling design. Sexual behaviours were assessed through a structured questionnaire. Multivariable analysis was used to identify association between various individual level characteristics and probability of engaging in sexual activities involving men.

**Results:**

Nearly one-third (29 percent) reported having had non-marital sex in their lifetime. Of these men 16 percent reported premarital sex, while 11 percent reported engaging in both pre- and extramarital sex. Only two percent reported exclusive extramarital sex. In total 211 respondents, 9 percent reported ever having had sexual relations with men. While 62 respondents, 2.6 percent reported exclusive sex with males. Factors that were significantly associated with MSM behaviours were being less than 27 years (adjusted OR 5.4, 95% CI 3.8–7.7, p < 0.000), less than 10 years of schooling (adjusted OR 2.1, 95% CI 1.4–3.2, p < 0.000), being unemployed (adjusted OR 2.7, 95% CI 1.6–4.3, p < 0.000), being exposed to pornographic materials (adjusted OR 4.8, 95% CI 3.0–7.7, p < 0.000) and being a migrant (adjusted OR 2.1, 95% CI 1.3–3.4, p < 0.002). Factors significantly associated with exclusive homosexual behaviour were having sexual debut at a younger age i.e. 16–22 years (adjusted OR 12.5, 95% CI: 3.8–40.7, p < 0.000), being unemployed (adjusted OR 8.8, 95% CI: 3.0–26.0, p = 0.000), having had exposure to pornographic materials (adjusted OR 3.3, 95% CI: 1.5–7.2, p = 0.002).

**Conclusions:**

To prevent the spread of STI’s in Pakistan, preventive interventions should focus on reaching out to young uneducated men offering them with appropriate counselling and skills to adopt “safe sex practices” through workplace orientation sessions; while for youth in schools, life skills education be included in the curriculum. Through public-private partnership stigmatised groups should be reached through established community networks and provided with information on accessing voluntary counseling and treatment centres.

## Introduction

Pakistan is currently the sixth most populous country in the world, with an estimated population of 180 million [1]. Pakistan has an estimated 97,400 people living with HIV at the end of 2009, with 2917 AIDS patients [2]. HIV prevalence in the general population remains under 0.1% [3].

Although, Pakistan has been identified as a low HIV prevalence country, available evidence shows that the country is already experiencing a concentrated epidemic in the high-risk groups such as the injecting drugs user (IDUs), female and male sex workers [4,5]. The infection has therefore, the potential to spread from these groups to the general population. UNAIDS has recently cautioned that the epidemic is growing rapidly in several South Asian countries including Pakistan, Vietnam, and Indonesia [6]. In the East Asia there are now reports of an increase in the numbers of new cases of HIV among men who have sex with men ‘MSM’ (MSM is an abbreviation used for ‘men who have sex men’ or ‘males who have sex with males’. The term ‘men who have sex with men’ describes males who have sex with males, regardless of whether or not they have sex with women or have a personal or social gay or bisexual identity) [7,8].

Recognizing the potential threat of a generalized HIV epidemic that might occur through men who engage in risky sexual practices, the National Aids Control Program of Pakistan (NACP) commissioned a pioneering behavioural and biological study of urban men funded by DFID and implemented by the Population Council in 2007. The study was commissioned with the primary objective to measure the prevalence of five selected sexually transmitted infections (that included syphilis, gonorrhoea, chlamydia, HSV-2 and HIV) [9]. The secondary objective was to measure sexual behaviours in urban men who could act as a bridge for spreading STIs from the high to low risk groups. This paper presents the reported results of men’s non-marital sexual behaviours with a focus on same-sex sexual activity.

## Methods

A cross-sectional household survey of 2400 men was carried out in six major cities of Pakistan (Lahore, Karachi, Peshawar, Quetta, Rawalpindi and Faisalabad) in 2007. In each city 400 men aged 16–45 years were selected through multistage sampling techniques. The sample size was based on a power calculation to deduct a significant decrease in sexually transmitted infection (STI) prevalence in five years and in terms of its power to detect behavioural change with statistical confidence [10].

Initially in each city 10 blocks, 2400 households in each block, demarcated by the Population Census Organisation were selected based on probability proportionate to socio-economic status using female literacy as a proxy indicator for economic status. Within each block, 40 men were selected who had been resident in the selected households (households comprised houses, hostels and apartments) on the night prior to enumeration through systematic random sampling. Informed consent was obtained, twice for administering a behavioural questionnaire and obtaining biological specimens.

The age band took into account the near-universal finding that men’s risk behaviour declines from about age 30 and reaches very low levels by age 50 [11]. The study population included both married and unmarried men from different occupations (including those working in the informal sector) as well as those who were students or unemployed. It represented most linguistic groups since migration to cities is likely to originate from almost all parts of the country.

### Data collection

The quantitative data were obtained by a study team that comprised two male social scientists. The questionnaire was based on the validated questions available in the Behavioural Surveillance Surveys-BSS [12]. The information collected on sexual behaviours included history of sexual debut including age and type of partner, sexual partners in the last 12 months and last three months, type of sex including anal, oral and vaginal sex, condom use ever and last time, and personal hygiene.

For data collection in each city two teams were hired locally. All teams were assembled in Islamabad and provided extensive training. Field work was carried out and completed in approximately 40 days from July 1 to August 10, 2007.

### Ethical approval

Ethical approval was obtained from the Human Research Review Committee of HOPE a Pakistan based research Organisation, the Institutional Review Board of the Population Council New York, and the Ethics Committee of the London School of Hygiene and Tropical Medicine, London. Informed consent was obtained from all study participants who were provided comprehensive information about the study. Participants were given four options to confirm their informed consent: consent by signature; consent by fingerprint; oral consent with a witness; or oral consent recorded by audiotape. In case of respondents aged 16 and 17 years, informed consent was obtained from their parents and guardians.

### Data analysis

Data entry was carried out using CSPro version 3.2 and data analysis was carried out by SPSS version 14. Statistical analysis included descriptive statistics, bivariate analysis using chi-square test and multivariable analysis using logistic regression models, with 95 percent confidence intervals.

## Results

This paper reports results that reflect the non-marital sexual behaviours of men in major urban centers of Pakistan, with a special emphasis on male to male sexual activity. The refusal rate was 37 percent which is well within the range of similar studies [13,14]. We anticipated relatively high rates of non-response due to the sensitive nature of the subject matter of the questionnaire, the biological testing requirement, and the known difficulty in locating men in urban settings [13].To reach the sample size, in total, 5995 households had to be approached. Table 1 provides the socio-demographic characteristics of the study respondents.

**Table 1:** Socio-demographic characteristics of respondents (N = 2400)

Age*	**Years**
Mean age	29.1 SD ± 8.6
Median age	27.0
**Education**	**Percent**
No Education	14.9
Up to secondary	53.2
Above secondary	31.9
**Employment**	
Unemployed	3.0
Student	16.8
Employed	80.3
**Marital Status**	
Unmarried	47.8
Married	51.8
Formerly married	0.7

### Sexual debut

Among the total sample of 2,400 men, 31 percent reported never experiencing marital or non-marital sexual relation (i.e., were virgins), while 42 percent reported having had their first sexual encounter with their wives. Twenty-seven percent (n = 653) reported having premarital sex [10].

Of the 653 respondents who reported having a premarital sexual encounter as their first sexual experience, 53 percent did so with a female other than a sex worker (FOSW- A sexual relationship that did not involve any monetary transaction) involving no monetary transaction, described by respondents as ‘friends’, 28 percent did so with a female sex worker, 3 percent with a male sex worker, and 17 percent with a male other than sex worker described by respondents as ‘friends’. Only 3 respondents, 0.5 percent reported having had sex for the first time with a *hijra* (in the South Asian context, the word Hijra is described as an umbrella term used for those men who are transgender, eunuch, transvestites, hermaphrodites or intersexed, bisexuals or homosexuals) [15].

### Age distribution at sexual debut

The mean and median age at the time of marital sexual debut was 24 years (Standard Deviation 4.808). The mean and median ages at the time of sexual debut for pre-marital sexual relation were 19 and 18 years, respectively.

#### Non-marital sexual behaviours over different periods

In order to determine the extent to which the study population engages in premarital or extramarital sexual activity, all respondents were asked to recall their non-marital sexual relation over their lifetime, within the last twelve and three months.

Nearly one-third (29 percent) reported having had non-marital sex in their lifetime. Of thesemen16 percent reported premarital sex, while 11 percent reported engaging in both pre- and extramarital sex. Only two percent reported exclusive extramarital sex. In total, 13 percent of men reported engaging in extramarital sexual activity.

**Figure 1:**
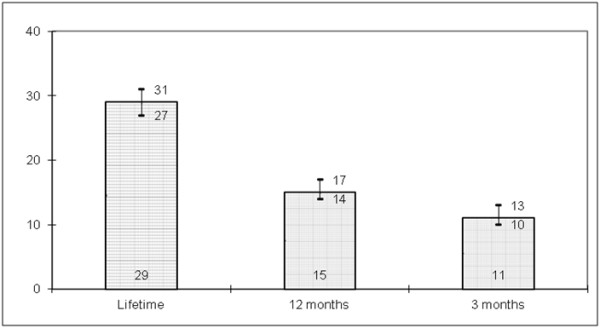
Percentage of men reporting non-marital sexual behaviour, over three time periods (with 95% CI).

Figure 1 shows non-marital sexual behaviour over different time periods (lifetime, last 12 months, and last 3 months) with 95 percent confidence intervals. 11 percent of respondents reported non-marital sex in the last three months, 15 percent in the last 12 months and 29 percent reported non-marital sex ever in their lifetime.

**Figure 2:**
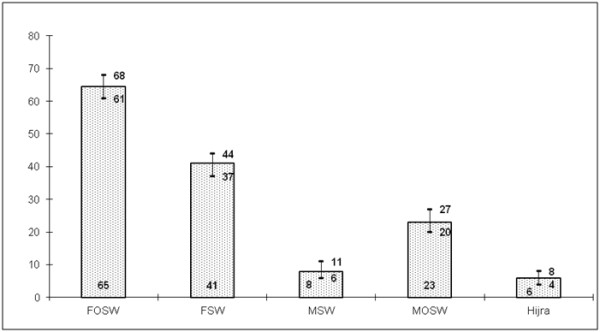
Type of sexual partners among men who reported non-marital sex with 95% CI (n = 697).

Among the respondents who ever had non-marital sex in their lifetimes (n = 697), as seen in Figure 2, 65 percent reported having sex with a female partner who was not a sex worker with no financial transaction being reported. These partners in most instances were labelled by respondents as ‘friends’. The next most frequent category was female sex workers (41 percent overall).The third most frequent non-marital sexual liaison was reported with males who were not sex workers (23 percent) while 8 percent reported sex with a male sex worker. Sex with hijra sex worker was about 6 percent in all six cities. Multiple responses were possible as respondents could have more than one partner. Our results show that overall 44 percent of those who reported non-marital sex said they had three or more non-marital partners in their lives while 14 percent reported two and 39 percent had one partner only.

**Figure 3:**
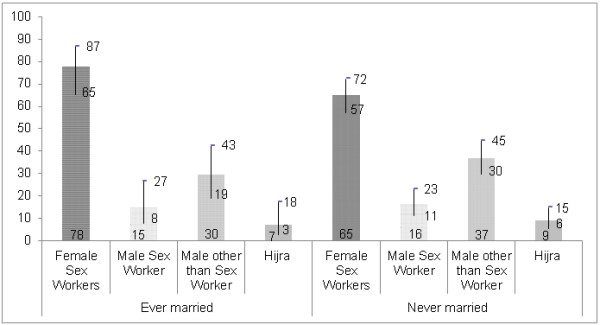
Type of high-risk sexual partner, by marital status (with 95% CI).

Looking at the distribution of high-risk partners within the married and unmarried categories of Figure 3 the results suggest that unmarried men had higher rates of sexual activity with male sex workers, males other than sex workers, and hijras as compared to married men. Most unmarried men were in the younger age category.

### Relationships involving men

In total 211 respondents (9 percent, 95% CI 7.7–10.0) reported ever having had sexual relations with men. Among these 26 percent were married. Out of these 62 respondents (2.6 percent, 95% CI 2.0–3.3) reported exclusive sex with males. Overall, the reporting of sexual relationships with men was significantly more likely among unmarried men as compared to married men (p value <0.000).

#### MSM activity in the last 12 months

**Figure 4:**
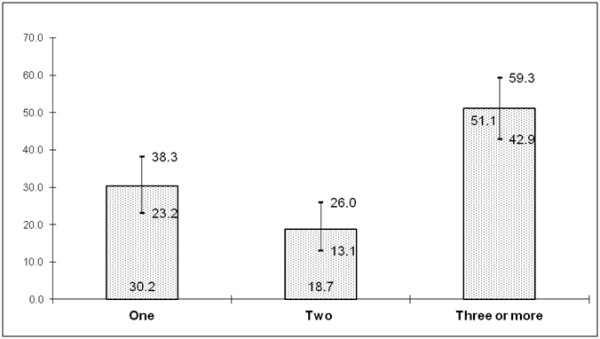
Number of partners of MSM in last 12 months (with 95% CI).

As shown in Figure 4 nearly half of the MSM men reported having had three or more sexual partners in the last 12 months.

#### Perception of risk of MSM men

Nearly half of the MSM respondents when enquired as to whether they considered themselves to be at risk of acquiring a sexually transmitted infection replied in the affirmative (p value <0.000).

#### Multivariable analysis of MSM and exclusive MSM activity

Multivariable analysis was carried out to identify the association between various individual level characteristics and the probability of engaging in sexual activities involving men. Two models (Tables 2 and 3) were constructed for the purpose. The first identifies the contribution of covariates to being sexually active for MSM while the second model explores the contribution of covariates to the probability of men having sex exclusively with men. The outcome variables in the first and second models were respectively based on 211 MSM (included 146 bisexual men as well 62 men having exclusive sex with men) and 62 exclusively homosexual men. As the first step, univariable analysis was carried out for each logistic regression model. The purpose was to determine individual association of each independent variable with the respective outcome variable. Thus an odds ratio and a 95% confidence interval was calculated for each associated factor. The likelihood ratio test was used for testing the significance of the coefficients. Independent variables with p-value of less than 0.25 were selected for the multivariable analysis.

**Table 2:** Significant factors associated with MSM activity among the sexually active men (n = 211)

**Variables**	**Adjusted OR**	**95% CI**	**P value**
Age*			
Up to 27 years	5.4	3.8 – 7.7	0.000
>27 years	1		
***Educational status***^**+**^			
Up to 10 years	2.1	1.4 – 3.2	0.000
>10 years	1		
***Occupation***			
Unemployed	2.7	1.6 – 4.3	0.000
Service (public or private)	1.8	1.2 – 2.8	0.005
Self Employed	1		
***Ever viewed pornographic materials***			
Yes	4.8	3.0 – 7.7	0.000
No	1		
***Migration status***			
Short-term Migrants^⊕^	1.1	0.7 – 1.7	0.585
Long-term migrants^⊕^	2.1	1.3 – 3.4	0.002
Non-migrants	1		

* 27 years was the median age of the respondents of the overall sample.

^+^10 years of education is the demarcation between secondary and higher secondary education. Educational attainment in seven cases was non-formal.

^⊕^Short-term Migrants are respondents who stayed away from their natal or marital homes for one night or more and Long-term.

Migrants are respondents who stayed away from their natal or marital homes for four months or more*.*

**Table 3:** Significant factors associated with men having exclusive sex with men among the sexually active population (n = 62)

**Variables**	**Adjusted OR**	**95% CI**	**P value**
***Age at sexual Debut***			
16-22 years	12.5	3.8 – 40.7	0.000
>22 years	1		
***Occupation***			
Unemployed	8.8	3.0 – 26.0	0.000
Service	4.5	1.5 – 12.9	0.006
Self Employed	1		
***Ever watched pornographic materials***			
Yes	3.3	1.5 – 7.2	0.002
No	1		
***Cigarette smoking***			
Yes	0.3	0.1 – 0.6	
No	1		0.001

Next, multivariable analysis was performed to look for the association of independent variables with the two outcome variables (in two separate models) while adjusting for the confounding effect of the other variables. In addition to the independent variables selected at univariable analysis, biologically plausible variables were also selected at this step. Significance of each independent variable in the multivariate analysis was assessed by its confidence interval and Wald statistic. Variables, which were not significant at a p-value of 0.05 or not considered biologically plausible were removed from the model. The overall significance of the variables in the model was assessed by the G statistic. Adjusted odds ratios and 95% Confidence Intervals were used for interpretation of the model.

Factors that had a significant association with men engaging in MSM behaviours were being less than 27 years (the median age of all respondents in the sample), having education less than 10 years (adjusted OR 2.1, 95% CI 1.4–3.2, p < 0.000), being unemployed (adjusted OR 2.7, 95% CI 1.6–4.3, p = 0.000), having ever viewed pornographic materials (adjusted OR 4.8, 95% CI 3.0–7.7, p = 0.000) and being a long-term migrant i.e. living away from natal or marital home for more than 4 months (adjusted OR 2.1, 95% CI 1.3–3.4, p = 0.002).

In Table 3, the factors significantly associated with exclusive homosexual behaviour were having sexual debut at a younger age i.e 16–22 years (adjusted OR 12.5, 95% CI: 3.8–40.7, p < 0.000), being unemployed (adjusted OR 8.8, 95% CI: 3.0–26.0, p = 0.000), having had exposure to pornographic materials (adjusted OR 3.3, 95% CI: 1.5–7.2, p = 0.002).

## Discussion

In Pakistan, sex outside marriage is completely proscribed by law^a^. Our study results, on the other hand, show that a sizeable minority of Pakistani men are indulging in sexual practices outside of marriage that are commencing at a young age and include sexual practices that have not been previously documented. The multivariate analysis has helped in identifying some of the factors associated with this practice. The significance level at 0.05 and low p value established that associates were unlikely to have occurred due to chance alone.

This study provides empirical evidence to describe the range and magnitude of non-marital sexual practices prevalent in Pakistani society. Our results are supported by other studies from South Asia that found relatively high rates of both extramarital and premarital sexual activity. Collumbien’s study in 2000among urban and rural men in India found that 25 percent had sex before marriage [16], and did so with a variety of partners (e.g., sex workers, friends, relatives, and future spouses). Faisel and Cleland, in 2006, found that 55 percent of the unmarried men in their study of migrant men in Lahore reported being sexually experienced [17].

Another important finding from the study is that it reveals exclusive male sexual activity with men (2.6% in our study). Our results conform to the range reported in some other Islamic countries. In the Middle East and North African regions, the prevalence of exclusive MSM activity in Morocco has been reported at 3.7% and in Sudan 2.0% [18,19].

We do acknowledge that our estimates of males having sex exclusively with males are possibly an underestimate. Most studies are believed to underestimate the prevalence of male-to-male sex [20]. The issue of male-to-male sex in most societies is associated with stigma making it difficult for people to discuss the issue candidly. For this reason we trained out interviewers to build rapport and establishing trust with the respondents in order to obtain information of a sensitive nature and assuring the confidentiality and maintaining privacy while conducting the interview. The findings of our study however have important implications for preventing and controlling the future spread of the HIV epidemic in Pakistan. At least 5 percent to 10 percent of all HIV infections in the world are transmitted through unprotected sex between men [20]. Population movements involving migrants, refugees, displaced persons and men employed in long-distance transportation or shipping help to drive the spread of HIV/AIDS and other sexually transmitted infections (STIs). Our results show that sex with males is more likely to occur at younger ages (16–20 years) and declines with age. At younger ages men often begin sexual activity, and their choice of sexual partner at this age is often based on availability and affordability and not necessarily on sexual orientation or preference. This may explain why male sexual partners are reported comparatively higher in the lower age groups than in the older ones. With a large cohort of Pakistan’s population in the adolescent age group an important recommendation from this study is that in addition to school health programs, special interventions must also be introduced that reach out of school youth who is employed in the informal sectors in order to educate them about safe sexual practices.

In 2006, more than 70 percent of HIV-positive people in South and Southeast Asia were men. In Pakistan, of the reported cases of those infected with HIV, men outnumbered women by seven times, and 7percent of HIV-positive individuals were men who have sex with men [21]. According to a recent study in Karachi and Lahore, 4percent of 200 male sex workers/hijras tested were found to be HIV positive [22]. Other findings and anecdotal evidence suggest that males engaging in sex with other males in Pakistan is becoming more commonplace [23,24].

As report in India, many men who have sex with men do not do so exclusively – they also have sex with women. Forty two percent of the respondents in a survey of MSM in Andhra Pradesh were married [25]. A sample of 482 men who had sex with men in Beijing found that nearly two-thirds also had sex with a woman, 28percent of them within the past six months [26]. In our study among the 211 MSM men, 26percent were married. This has important implications for the potential for an HIV epidemic in Pakistan as married men can act as the bridge to spread the infection from the high risk groups to the general population. Sex between men may take place because it is what is immediately available, for example in prisons or among long distance truck drivers [27,28]. Men who engage in it also may not identify themselves as homosexual and in other situations will have sex with women.

Across Asia and the Pacific, governments are putting more money and resources into HIV prevention and AIDS treatment, but few countries have addressed the entirety of the challenge. Among 20 Asia-Pacific countries surveyed in 2006, only nine of the National Strategic Plan on AIDS had included MSM and HIV specific programmes or interventions, such as peer outreach despite that Asia’s AIDS pandemic first appeared among MSM [20]. Only eight countries had any form of surveillance specific to MSM, while only five reported the inclusion of MSM men in Behavioural Surveillance Surveys [29]. In Pakistan, based on the empirical evidence provided by our survey, reaching out to MSM must be recognized as an important preventive strategy. Community networks organized by Non-governmental Organisations (NGOs) can play an important role in reaching out to such groups [30].

In describing their sexual relationships a high proportion of respondents in our study reported relationships with “friends” who were either males or females, with no financial transaction taking place in exchange for sex. However this phenomenon needs to be more deeply understood as it is not known whether these “friends” also had concurrent partners.

Sexual partners labelled as “friends” have also been reported in the earlier study by Faisel and Cleland [17].The concept of unpaid sex with these friends needs further elaboration. Having multiple sexual partners enhances the risk of acquiring STIs and should therefore be a focus of future IEC campaigns that focus on high risk practices.

Another finding from our study shows that living away from their natal or marital home predisposes men to risky sexual practices. These findings are supported by the literature that argues that mobility due to occupation is a risk factor for STI transmission [31]. It is possible that men living away from their families are free from socio-cultural and familial constraints and hence have greater opportunities to engage in non-marital sex.

### Conclusions and recommendations

Non-marital sex is not uncommon in Pakistan; and it takes place between a range of partners, including same-sex ones. Based on our findings we recommend a holistic strategy that will reach out to young adults and will include both the creation of awareness as well as service provision for preventing the spread of STIs in society. As the first step, we recommend taking on-board parliamentarians and introducing legislation that will facilitate the provision of Sexual and Reproductive Health (SRH) Services at all facilities under the government’s Health and Population Welfare Departments, and to all individuals irrespective of their marital status.

For this to happen, there is a need to develop a uniform SRH curriculum and impart training regarding it to all existing health providers. This training must include specific modules on youth counselling and the importance of quality of care. Aahung (a local organization working in sexual and reproductive healthcare in Pakistan) has already developed a curriculum for medical schools; this should be scaled up to include all medical colleges in the country. A national media and communications strategy should also be developed to raise awareness amongst the youth and to sensitize them on preventive aspects regarding healthy living and safe sex practices and to inform them about where they can obtain help and guidance on these matters.

Out of school young adults must be approached through workplace health education services. Those adolescents who attend school should be taught life skills education, which is incorporated into the school’s curriculum. Public sector providers already working in the existing Voluntary Counselling and Treatment (VCT) centres should reach out to the vulnerable and stigmatized populations through a public-private partnership approach. This can be done via community networks and groups established by the private sector, such as the Pakistan Society, Infection Prevention Society, Nai Zindagi etc., and they can provide information on practicing safe sex, along with counselling and management options.

### Study limitations

We acknowledge that self-reported information, particularly related to sexual behaviour can be subject to recall bias [32]. A meta-analysis of 28 studies, investigating the optimal length of recall period for self-report of sex and drug use behaviours, examined the reliability of three recall periods 1, 3 and 6 months. All three recall periods demonstrated acceptable reliability. For most sex behaviours, a recall period of 3 months was found to be most reliable, while 6 months was best for recalling number of sex partners [33].

As our study design was descriptive, analytical and cross-sectional; therefore, it does not allow for determination of causality related to risk behaviours and exposure to specific interventions or to identify trends.

## Endnotes

^a^According to Pakistan Penal Code (Act XLV of 1860), under section 496-B, a man and a woman not married to each other are said to commit fornication if they wilfully have sexual intercourse with one another. They shall be punished with imprisonment for a term which may extend to five years and also be liable to fine.

## Abbreviations

CI: Confidence interval; DFID: Department for international development; FOSW: Female other than a sex worker; HIV: Human immunodeficiency virus; HOPE: Health oriented preventive education; MSM: Men who have sex with men; NACP: National aids control program; OR: Odds ratio; VCT: Voluntary counselling and testing.

## Competing interests

The authors declare that they have no competing interests.

## Authors’ contributions

AMM was involved in conceptual design for this study, interpretation of data, drafting of the manuscript and critically revising for intellectual content. AW was involved in design of conceptual structure. He later contributed to data analysis, interpretation and was involved in drafting the paper. SP was involved in assisting with data analysis and in the critical revision of this article. MK was involved in acquisition of data and carried out analysis for the report. IM was performed all statistical analysis on SPSS and helped in generating the tables and graphs. All authors read and approved the final manuscript.
